# Polyvinyl Alcohol, a Versatile Excipient for Pharmaceutical 3D Printing

**DOI:** 10.3390/polym16040517

**Published:** 2024-02-14

**Authors:** Nadine Couți, Alina Porfire, Rareș Iovanov, Andrea Gabriela Crișan, Sonia Iurian, Tibor Casian, Ioan Tomuță

**Affiliations:** Department of Pharmaceutical Technology and Biopharmacy, Faculty of Pharmacy, University of Medicine and Pharmacy “Iuliu Hatieganu”, 400012 Cluj-Napoca, Romania; nadine.couti@elearn.umfcluj.ro (N.C.); riovanov@umfcluj.ro (R.I.); crisan.andrea@umfcluj.ro (A.G.C.); sonia.iurian@umfcluj.ro (S.I.); casian.tibor@umfcluj.ro (T.C.); tomutaioan@umfcluj.ro (I.T.)

**Keywords:** polyvinyl alcohol, pharmaceutical excipient, 3D printing, fused deposition modeling, hot melt extrusion

## Abstract

Three-dimensional (3D) printing in the pharmaceutical field allows rapid manufacturing of a diverse range of pharmaceutical dosage forms, including personalized items. The application of this technology in dosage form manufacturing requires the judicious selection of excipients because the selected materials must be appropriate to the working principle of each technique. Most techniques rely on the use of polymers as the main material. Among the pharmaceutically approved polymers, polyvinyl alcohol (PVA) is one of the most used, especially for fused deposition modeling (FDM) technology. This review summarizes the physical and chemical properties of pharmaceutical-grade PVA and its applications in the manufacturing of dosage forms, with a particular focus on those fabricated through FDM. The work provides evidence on the diversity of dosage forms created using this polymer, highlighting how formulation and processing difficulties may be overcome to get the dosage forms with a suitable design and release profile.

## 1. Introduction

By providing an adaptable platform for the fabrication of therapeutic products that can easily adjust to shifting patient demands and market conditions, three-dimensional (3D) printing holds the potential to completely transform the production of pharmaceuticals intended for oral administration routes. Large-scale conventional manufacturing processes (such as tableting and encapsulation) do not offer a platform for dose flexibility, while smaller-scale manufacturing processes (such as manual capsule filling) present difficulties due to high labor costs and solubility problems. Overcoming these obstacles and creating dosage forms for pre-clinical and clinical trials more quickly, efficiently, and cheaply could all be possible using 3D printing [1]. It has been demonstrated that one of the most promising printing methods for producing medicinal products is fused deposition modeling (FDM) [2]. Scott Crump created the FDM technique in 1989. Some years later, Crump and his wife co-founded Stratasys, which went on to commercialize the first FDM printer. These days, FDM is the most popular 3D printing technique in the pharmaceutical industry [3]. During FDM, the printing nozzle heats the filaments beyond their components’ glass transition temperature, causing them to deposit in multiple layers with adjustable height and dimension on a heated flat surface. Filaments used in FDM are composed of plastic polymers. A dosage form that is FDM-3D-printed can have its makeup, release kinetics, design, and size precisely tailored to the patient’s needs [2]. Nevertheless, there are certain disadvantages as well that restrict the use of FDM in pharmaceutics. The absence of laws pertaining to design creation, manufacturing process, and quality testing is a significant drawback of additive manufacturing [4]. A block in the printing head can often occur and may result from the filament breaking in circumstances when it is too fragile to withstand the mechanical strain created by the pressure of pushing [5]. Other obstacles in creating FDM 3D-printed tablets are the slow release of the medicine, low drug–polymer miscibility, poor printability of the polymers employed, and high processing temperature [6]. Due to heat degradation during both HME and FDM, there has been an association with a decrease in drug contents. Consequently, it is crucial to reduce the risk of drug degradation by decreasing the processing temperature [3]. Another drawback is that a small percentage of pharmaceutical-grade polymers have the necessary thermoplastic properties to be used in FDM printing [7]. The most used polymers in 3D printing have the advantage of their adaptability, low cost, and convenience. Among these polymers, polyvinyl alcohol (PVA) is becoming more and more popular because of its suitable flowability, biodegradability, and affordability [8].

For many years, the pharmaceutical and medical industries have used physically or chemically modified PVA structures [9]. Firstly, the PVA hydrogels and films were used for medicinal applications [10]. Then, PVA’s disintegrant and lubricant properties were discovered and exploited [11]. In the 3D printing processes, PVA has been used as a support material for a long time, which is why several pharmaceutical sorts were developed [8]. During the last few years, PVA has made its mark as a filament-forming excipient for FDM. Since solid oral dosage forms are relatively easy to fabricate and handle and have a high patient compliance rate, they have long been the favored way of medication administration. In addition, because they are so simple to deliver, healthcare personnel are typically not needed [3]. A significant number of studies have focused on inspecting PVA’s printability when combined with different types of drugs, on analyzing the stability of the printed forms, or on many other aspects, like the geometry of the printlets, multidrug-containing dosage forms, disease-specific tablets, and batch traceability [12,13,14,15,16]. The purpose of this review was to evaluate the developments regarding the use of PVA in hot melt extrusion (HME) coupled with FDM to print solid oral dosage forms containing one or more active pharmaceutical ingredients (APIs).

## 2. Short History and Applications of PVA

The synthetic water-soluble resin with the largest volume is PVA, a polyhydroxy polymer [17]. PVA is considered to be one of the earliest synthetic polymers, but despite this, it continues to garner significant interest in the chemical, material, and medical fields due to a unique combination of its properties, which include water solubility, film orientation features for a liquid crystal display polarizer, adhesive capacity to a variety of substrates, nontoxic nature, biodegradable properties, and biocompatibility [9]. Hermann and Haehnel produced PVA for the first time in 1924 by saponifying poly(vinyl ester) in sodium hydroxide solution [18]. In 1927, the first academic studies on PVA were released. PVA is primarily used in the production of poly(vinyl butyral), fibers, protective colloids for emulsion polymerization, textile sizing, adhesives, and paper sizing. In addition, large quantities are used to make insecticides, herbicides, fertilizers, water-soluble films for containment bags for hospital laundry, and joint cement and additives for concrete used in the construction of buildings. Lesser amounts are used as emulsifiers for photo-printing plates, soil binding to prevent erosion, and temporary protective film coatings [17].

Since 1955, PVA sponge has been used as an arterial substitute in the medicinal field [19]. PVA is also frequently used in medicine as a tissue adhesion barrier, embolization particle, soft contact lenses, eye drops, artificial cartilage, and meniscus [20].

Solid scientific data support PVA’s safety. At concentrations up to 10%, it is non-irritating to the skin and eyes; cosmetics manufacturers prefer quantities up to 7% [11]. Many cosmetic applications use partially hydrolyzed grades of PVA because of their emulsifying, thickening, and film-forming properties [17]. Since it produces an occlusive and tensor action, PVA is one of the fundamental ingredients in the majority of peel-off masks [21]. A recent investigation combined a PVA-containing aloe vera-based polymeric membrane with green clay via green nanotechnology to develop a peel-off mask with many beneficial properties and further attainable applications in skin diseases. PVA is known to be one of the few vinyl polymers that can exhibit swelling behavior with an increased rate of biodegradation. This is feasible because of the hydroxyl groups which influence the material’s hydrophilic character [10].

PVA has been used in medicinal products for decades [17]. PVA is primarily utilized in oral preparations in the pharmaceutical field. Crosslinked PVA was evaluated as a potential disintegrant by Patel et al. The study found that this particular PVA had exceptional tableting properties, good flow, and outstanding disintegration activity [22]. A swelling controlled release system with PVA and emedastatine was evaluated by Morita et al., and a zero-release order kinetics was found [23]. Since 1990, several polymers, such as hypromellose, pullulan, chitosan, and pondac, a copolymer of PVA and methacrylates, have been proposed for use in the production of capsules. Adsorbing onto the crystal surface, polymers such as PVA can change the etching patterns of acetaminophen, for example, which in turn affects the rate of dissolution [24]. As a lubricant, PVA has been utilized in transdermal patches and sustained-release formulations for oral administration [11]. When creating solid dispersion formulations, poorly water-soluble drugs can be dissolved using PVA, which can develop a micelle-like structure. Being a non-ionic, pH-independent polymer, PVA can exhibit consistent solubilization performance throughout the gastrointestinal tract, resulting in minimal variability and elevated bioavailability [25]. Ophthalmic dosage forms represent other marketed pharmaceutical products that rely on PVA’s distinctive characteristics. PVA is a suitable ophthalmic vehicle for an array of preparations owing to its inferior viscosity and surface tension and its superior adhesive qualities. Summed up, these characteristics favor the fabrication of a transparent film that functions as an excellent optical system [26]. For ophthalmic solutions, which are viscous formulations, PVA is employed as a viscosity-increasing component. Artificial tears and contact lens solutions rely on its lubricant properties [11]. PVA also plays a crucial role in the formulation of polymeric ophthalmic inserts, especially those containing pilocarpine, like Ocusert [27]. Another ophthalmic drug delivery system that was developed and marketed is Vitrasert, manufactured by Bausch & Lomb and approved in 1996, a ganciclovir cylindrical core enclosed in an ethylene vinyl acetate cup with permeable PVA membranes on one or two surfaces to facilitate diffusion [28].

Liposomes are an ideal colloidal drug carrier system for intravenous injection that can integrate or be associated with a wide range of medicines. One way to alter the liposomes’ surface is through polymer coating [29]. During the last ten years, the coating of liposomes with mucoadhesive polymers has been developed and used. Chitosan, polyacrylates, and long-chain PVA are usually employed for this purpose [24].

In order to create diverse membranes and hydrogels, PVA has frequently been utilized. PVA gels exhibit excellent biocompatibility with tissues, bodily fluids, and blood while also causing very minor mechanical irritations [10]. PVA hydrogels are a preferred material for a variety of pharmaceutical uses, such as controlled drug release, due to their adaptable qualities. One more feature of PVA that makes it the preferred polymer for drug-release applications in topical products is its bioadhesive property. The degree of cross-linking, pore size, and type of integrated drug all influence the drug release rates from PVA hydrogels, which usually adhere to first-order kinetics [30].

When first introduced in 3D printing processes, PVA was used as a water-soluble support structure for PETG, PLA, and nylon construction materials [31]. There have been many developments in the field of FDM-fabricated oral dosage forms in the last years. PVA may be a good fit for a number of biomedical pharmaceutical uses, especially when it comes to FDM’s development of sustained-release drug delivery systems. Additionally, PVA can be used for drug combinations with immediate release for an ideal absorption and for floating dosage forms [32].

## 3. PVA Chemistry

**Figure 1:**
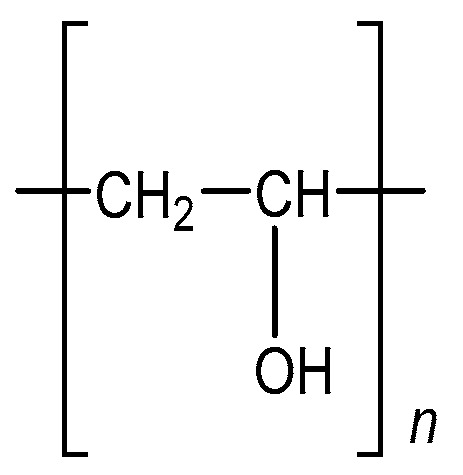
Chemical structure of polyvinyl alcohol.

PVA is the synthetic water-soluble polymer defined by the formula (C_2_H_4_O)n. Chemically, PVA is considered an ethanol, or vinyl alcohol, homopolymer. The structural formula of PVA can be seen in Figure 1 [33].

Since vinyl alcohol and acetaldehyde are isomeric, vinyl alcohol cannot be polymerized directly. As a result, PVA is frequently produced by polymerizing a protected monomer, like vinyl ester, and then deprotecting it [9].

**Figure 2:**
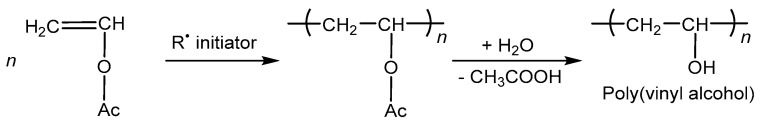
Radical polymerization of vinyl acetate to create PVA [9].

PVA is commercially manufactured by hydrolyzing poly(vinyl acetate) (PVAc), in two steps: first, vinyl acetate is free radical polymerized to PVAc, and then PVAc is hydrolyzed to produce PVA, as shown in Figure 2 [9,34].

The properties of PVA resin depend on both its degree of polymerization (DP, or n) and its degree of hydrolysis (DH), which determines the percentage of hydroxyl groups on the structural framework. Lesser hydrogen bonding results from lesser DH and a higher percentage of remaining acetate groups, which reduces stereoregularity and crystallinity [35]. Commercially available materials have a value of n (degree of polymerization) between 500 and 5000, which corresponds to a molecular weight range of roughly 20,000–200,000 [11]. The degree of hydrolysis for commercially accessible fully hydrolyzed grade PVA usually ranges between 98 and 99%, and for partially hydrolyzed PVA, between 80 and 98%. The polymerization degree for the fully hydrolyzed PVA is between 200 and 3000 [9]. Based on the viscosity of a 4% aqueous solution at 20 °C, PVA can be classified as low viscosity grade (4–20 mPa.s—PVA with DP of approximately 500, molecular weight around 20.000), medium viscosity grade (21–33 mPa.s—PVA with DP of approximately 3250 and a molecular weight of about 130.000), and high viscosity grade (40–65 mPa.s—PVA with DP around 5000 and a molecular weight of approximately 200.000) [36].

**Figure 3:**
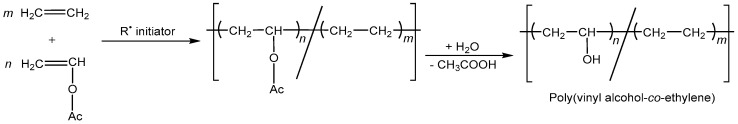
Poly(vinyl alcohol-co-ethylene) synthesis.

In addition to PVA homopolymers, a range of PVA-related compounds are manufactured industrially and are available for sale in a variety of grades. Copolymerization is a beneficial technique to create a new polymeric material with distinct features. Ethylene-vinyl alcohol copolymer, which combines high gas barrier qualities with low permeability, is typically produced by copolymerizing vinyl alcohol with ethylene and then hydrolyzing it, as shown in Figure 3 [9].

## 4. PVA Physical Properties and Pharmaceutical Grades

PVA presents itself as white to cream-colored granules or powder with no odor. The 4% water solution has a pH between 5 and 8 [17]. The dissolution of PVA in water is carried out by heating an aqueous dispersion obtained at room temperature, at about 90 °C for about 5 min. Additionally, further mixing is an important parameter, being used during dissolution but also during cooling of the solution [11]. Regarding water solubility, the lower the degree of hydrolysis (about 80–85%), the higher the solubility of PVA and the simpler it is for it to crystallize. Only heated to boiling water can completely dissolve fully hydrolyzed PVA. Although grades with a hydrolysis of 70–80% are only soluble at water temperatures of 10–40 °C, grades with a lower hydrolysis are soluble at ambient temperature. When the degree of hydrolysis increases, so does the viscosity, which reduces as the temperature rises [17]. With an increase in the degree of hydrolysis, the viscosity scale shifts from 3.4–52 to 4.0–60.0 mPa·s. Little additions of lower molecular weight aliphatic alcohols, urea, or salts like thiocyanates can stabilize viscosity to some extent. PVA grades that have undergone partial hydrolysis have more stable solution viscosities. When hydrolysis rises, resistance to organic solvents also increases [17].

Fully hydrolyzed PVA’s glass transition temperature for high molecular weight material was found to be 85 °C [17,32]. The melting point of PVA is between 180 and 220 °C. When PVA is partly hydrolyzed, its melting point can reach 180 °C; once completely hydrolyzed, it can reach 220 °C [32]. PVA softens at about 200 °C, it decomposes at about 228 °C [37]. PVA’s tensile elongation is highly responsive to humidity, varying from 10% when fully dry to 300–400% at 80% relative humidity. Plasticizer addition can increase these values by two-fold [17]. Different concentrations of a low molecular weight PVA (less than 6000 g) with a hydrolysation of 80% were developed by Babaie et al. No discernible shear-thinning activity in this concentration range was seen in diluted PVA solutions [38].

PVA-based excipients have been described and introduced to the market for oral sustained release and solubility enhancement. The compendial grade PVAs meet the requirements of all three major pharmacopeias (USP, Ph. Eur., and JPE). Pharmaceutical-grade PVAs are materials that have been partially hydrolyzed and are generally categorized using a coding system. The degree of hydrolysis is indicated by the first number after a brand name, while the second set of digits shows the approximate viscosity (dynamic), in mPa·s, of a 4% *w*/*v* aqueous solution at 20 °C [11,39]. The pharmaceutical grade polymers currently used in HME and FDM 3D printing applications are listed in Table 1, below.

Two more vinyl alcohol polymers were used in HME coupled with FDM to 3D print oral dosage forms, not as filament-forming polymers, but as coating agents. Kollicoat^®^ IR, a vinyl alcohol and ethylene glycol graft copolymer, is used for coating instant-release tablets. The same polymer can be utilized in sustained-release coatings as a pore-forming excipient, to build films, act as a protective colloid, or to stabilize suspensions and emulsions [51]. OPADRY^®^ II, a PVA-based high performance film coating system, was also used for coating pharmaceutical solid dosage forms with an aqueous film [52].

## 5. The Use of PVA in 3D Printing Technology

Due to its relatively low melting temperature, which results in a melt viscosity that is both sufficiently high for construction yet low enough for extruding, PVA is one of the thermoplastics preferred for additive manufacturing using FDM [53]. The production of feedstock containing the active pharmaceutical ingredient (API) in the form of a printable filament comes before the additive manufacturing [54]. Loading the active substance into filament by passive diffusion, a method used prior to HME, is still being used in some cases as it overcomes some of the HME-associated issues, mainly the thermal processing of the drugs. But, one of the principal reasons why there has been a shift away from this approach in recent years and towards HME is because lower drug loadings have typically been achieved in comparison to those obtained by HME. There is also a lack of easily accessible information regarding how the passive diffusion strategy may be used to optimize drug loading. Passive diffusion can currently only be used with highly potent medications due to the low loading, which is, in most cases, less than 1–2% API [55].

Most scientists have chosen the HME approach for the production of feedstock since it allows them to alter the filament’s composition as needed [54]. Physical blends of the API and excipients are thermally processed in this method, and then the molten mass is extruded through a die and solidified into filament form by cooling. The ability to prepare filaments with high drug loading is HME’s main benefit [13].

The first versions of FDM 3D printing, a type of additive manufacturing, appeared in the 1980s. During the FDM process, the printhead travels, and the heated mixture is discharged, constructing the prototype in thin layers. A temperature-controlled 3D printing nozzle with a specific diameter pushes the modeling material, in this case, the melted filament, into a construction platform at a pre-adjusted speed. The first layer is produced by the printer nozzle moving in the X–Y plane according to a specific pattern dictated by the shape of the object and its dimensions. By moving the nozzle or the platform through the Z plane at a distance equal to the layer thickness, successive layers are printed. Since the platform temperature is often lower than the extrusion head temperature, the printed material can solidify between the deposition of each layer. A pre-designed software data file coding a 3D object is primarily transformed into an actual item by adding successive layers of molten or semi-liquid modeling material. PVA, polylactic acid (PLA), and polycaprolactone (PCL) polymers, for example, have sufficient thermoplasticity to be used in FDM 3D printing [56].

### 5.1. Rheological Properties of PVA and Their Control

One of the fundamental issues with the HME process is establishing the necessary rheological properties, in order to assure the printability of the feedstock filaments, in addition to the drug’s thermal stability. The viscosity of the heated mixture has an influence on both HME and FDM steps [13].

In HME, the determination of melt flow rate is a quick screening method to evaluate the melt viscosity of the feedstock material [57]. Boetker et al. found that complex viscosities between 10^3^ and 10^4^ Pa⋅s could be the most appropriate for HME, ensuring the printability of the filaments [58].

In binary systems comprising Parteck MXP pharmaceutical-grade PVA polymer, 50% of the aforementioned polymer is required to make posaconazole’s viscosity in its melting temperature range adequate for HME. The value of this blend’s viscosity was between 10^3^ and 10^4^ Pa⋅s, in the suitable range that was also determined by other researchers beforehand. [59].

On account of the fact that there are few studies regarding direct viscosity measurements, researchers concentrated on assessing the extrusion process’s torque to indirectly determine the blend’s viscosity. Digkas et al. prepared filaments with diclofenac sodium as API, PVA as filament-forming polymer, several plasticizers (mannitol, erythritol, isomalt, maltodextrin, and PEG) and superdisintegrants (crospovidone and croscarmellose sodium). The torque value, measured in Nm, is a significant parameter that helps characterize the energy consumption of the extruder’s motor when processing filaments through HME. The nature and concentration of the plasticizers that were used, while maintaining consistent processing conditions for temperature, screw speed, and feed rate, largely determined how much energy was used during the extrusion process. When compared to extrudates containing 10% *w*/*w* plasticizer, those containing 15% *w*/*w* plasticizer showed lower torque values. The explanation is that higher plasticizer concentrations can lower the formulation’s melt viscosity inside the screw channel, which lowers screw stress and, in turn, lowers torque. Furthermore, the kind of plasticizer used did not noticeably affect the torque and mechanical energy. Nonetheless, filaments containing crospovidone showed improved processability compared to those with croscarmellose sodium [60]. Palekar et al. discovered that the processing torque and temperature needed for neat PVA were significantly higher than those needed for PVA containing sorbitol, indicating that neat PVA was challenging to process. Extrusion of clean PVA at lower temperatures (160–170 °C) showed noticeably higher torque (>77%), which caused the extruder to automatically stop. This may be explained by PVA’s high melting point and high melt viscosity. The extrusion was performed at a temperature more than 180 °C in order to treat neat PVA. The processing torque was dramatically lowered to 47–51% [61]. 

Researchers have emphasized how important rheology is to the success of FDM, presenting novel approaches to evaluate and optimize filament viscoelastic characteristics for improved printability and functional performance of printed formulations. One study devised a sensitive technique based on oscillatory shear rheology and mechanical analysis to evaluate the viscoelastic behavior of HME filaments, identify the best printing parameters, and forecast their effects on the printlets. For this, two distinct FDM 3D printers, Makerbot Replicator Mini+ and Voolt 3D model Gi3, were utilized to print PVA filaments with various plasticizer concentrations. PVA filaments containing 10 and 20 percent glycerol exhibited the appropriate viscosity behavior to ensure sufficient flow via the printer nozzle. The complexity of the PVA–glycerol 40% formulation’s viscosity suggests that the material’s strong malleability could jam the printer, preventing the filament from being fed in a constant rate to the nozzle. These results show how rheological modifications to the printing process have the capacity to alter or even obstruct it [62] The main limiting parameters to guarantee the printability of filaments, according to the results of Ilyes et al., are brittleness and melt viscosity. Temperature-dependent phase transformation controls the flow rate in both HME and FDM, and shear stress produced by the screw configuration in HME contribute to this process. It has been shown that the addition of APIs can generate lower complex viscosities compared to placebo filaments. While it is generally agreed upon that the processability interval for HME is between 800 and 10,000 Pa⋅s complex viscosity at 0.1 rad/s angular frequency, this study indicates that the following FDM 3D printing of dosage forms can operate in the range of 100 to 64,000. Based on these findings, the authors concluded that blends with complex viscosities up to 1200 Pa⋅s at 100 rad/s can still be printed, with values over 600 Pa⋅s heavily reliant on the shear-thinning potential and flexural modulus [63]. Pinho et al. evaluated the customized preformulation methodology that included physicochemical evaluations, especially rheological profiles of the samples, to direct the development of dosage forms using FDM 3D printing. For this, the thermolabile model API, isoniazid, was employed to analyze the polymers widely used in FDM printing, namely high impact polystyrene, PLA and PVA, as well as their usual plasticizing agents (mineral oil, triethyl citrate, and glycerol, respectively). The use of plasticizers in the PVA formulation had favorable effects on the solubility of drugs and rheologic behavior, likely enhancing the ability to print the sample [64].

In FDM, the filament may start to leak or flow before pressure is applied if the viscosity is too low. This phenomenon should be avoided because it could lead to poor prints and material waste [65]. As a result of the melt elasticity, the filament expands after leaving the die, causing die swell, an extensional rheology phenomenon of the polymer. Die swell that occurs at the nozzle’s end in FDM has the potential to warp the printed layer and compromise the drug’s dosing homogeneity. Altering the nozzle temperature or the ratio of the print head speed to the feeding speed can be used as a compensatory measure [4]. Shear viscosity is the most important rheological property and is dependent on both the internal factors such as the molecular structure, molecular weight, and molecular weight distribution of the polymer; the solid form of the drug; and the external factors such as the temperature and the shear rate. Shear rate at the printer liquefier depends on its dimensions and the printing speed, and because of the narrow nozzle diameter, it is typically very high, being well in the shear thinning region for most polymers [66].

Other studies referring to the addition of plasticizers have been conducted in relation to the FDM process. Sorbitol was found to be an effective plasticizer that lowered the glass transition temperature and made extrusion and printing easier. The complex viscosity of several formulations was compared to a commercial PVA filament by Ilieva et al., in order to assess the printing quality of each filament. Sorbitol percentages varied in formulations: 11%, 13%, and 15%. The 15% sorbitol formulation exhibited a markedly increased shear-thinning tendency, indicating that the addition of more than 13% plasticizer significantly alters the polymer melt characteristics, resulting in an overplasticized filament. The best placebo formulation was the one with 11% sorbitol, which virtually matched the commercial PVA filament’s complex viscosity. The complex viscosity was decreased by adding the API, paracetamol, to a 11% sorbitol formulation, but it still maintained its shear-thinning pattern, comparable to that of the commercial PVA filament [67]. Wu et al. improved the processability of PVA in FDM. The melting point of PVA was decreased and made appropriate for FDM by adding water, glycerol, and non-toxic plasticizers. It was discovered that a 50/50 blend of glycerol and water was the best combination for efficient FDM processing. A nozzle temperature of 175 °C was found to be the ideal printing parameter [68].

### 5.2. PVA as Filament-Forming Excipient for FDM 3D Printing

PVA represents one of the most used filament-forming thermoplastic polymers for the 3D printing of oral solid dosage forms. The range of pharmaceutical forms obtained using this polymer is diverse, starting from the simplest ones, such as immediate release cylindrical tablets, to specially designed forms, such as abuse-deterrent egglets, caplets, orodispersible tablets, and gastric floating tablets. As seen in Table 2, more and more researchers focus on 3D printing of solid oral dosage forms containing PVA as a thermoplastic excipient in order to cater to patients’ specific needs.

#### 5.2.1. Immediate Release Tablets

A considerable number of studies focus on producing immediate release tablets via FDM. Some of the scopes of these published works were to evaluate the processability of various APIs during the extrusion or the 3D printing phases, to compare different excipients, to analyze the advantages or disadvantages of several particle sizes of the same polymer, to come up with a model that predicts the drug’s release, or to facilitate batch traceability for a future in which 3D-printed tablets would be manufactured on a large scale.

Pereira et al. discovered that the method of employing distilled water as a temporary co-plasticizer reduced the temperatures for extruding (to 90 °C) and 3D printing (to 150 °C) from 170 °C to 210 °C, respectively, with a corresponding decrease in the stresses imparted to the chemicals. They created one form of uni-layer polypill with a rapid gastric release in addition to two types of multilayer polypills comprising lisinopril dihydrate, indapamide, amlodipine besylate, and rosuvastatin calcium. Parteck MXP was used as the matrix-forming polymer. They found that the four medication ingredients, during their joint processing at 90 °C, seldom interacted chemically with one another [69]. To focus on the processability of PVA, immediate release tablets with carvedilol or haloperidol as APIs, Parteck^®^ MXP as matrix-forming polymer and sorbitol as plasticizer were created by Wei et al. Filaments with 10% and 20% drug content needed temperatures between 180 °C and 190 °C for extrusion. This temperature requirement was lowered to around 150 °C by incorporating 10% sorbitol. Nevertheless, a printing temperature of 210 °C was still necessary for proper miscibility. The study concluded that PVA is not miscible with PEGs and poloxamers. Another inference drawn was that water-soluble compounds might not encounter problems related to drug–polymer miscibility when it comes to PVA [70]. Crișan et al. developed immediate release tablets with 10% to 60% diclofenac sodium, under Quality by Design (QbD) guidance. The mechanical characterization of the filaments revealed that a larger drug content, particularly in the case of 60%, hindered printability. Among the various tablet architectures tested, the honeycomb structure emerged as the most proper choice for customizable IR dosage forms. A predictive method was additionally created to optimize printing parameters, hence simplifying the creation of customized tablets [13]. Tablets containing indomethacin and Mowiflex^®^ C17 pulverized granules were created by impregnation and HME, and the quality of the tablets obtained by these methods was compared by Thanawuth et al. The resulting 3D-printed oral dosage forms were examined for various properties, including drug content and release. The study found that the drug loading method greatly impacted drug content and affected drug release. Specifically, tablets obtained through HME and impregnation with a lower drug content released the drug faster, while higher drug content in HME-produced tablets delayed release [71]. Oral dosage forms with timapiprant and various polymeric carriers were prepared by Uboldi et al. to test the printability of the carriers. A Gohsenol EG 03P-based formulation was found to be the most suitable because of its ability to maintain the API content, its good printability, and dissolution performance. These prototypes met the dissolution requirements and demonstrated comparable performance to the approved clinical phase III tablet product [72]. The PVA particle size was the subject of a study conducted by Crişan et al. Immediate release tablets with 30% ketoprofen and two kinds of partially hydrolyzed PVA, Mowiol^®^ 4-88 and Parteck^®^ MXP, respectively, were produced. In order to achieve the rapid release of the API in a PVA-based formulation, it is vital to minimize the residence time during the HME process by choosing polymers with small particle sizes and guaranteeing proper flow characteristics [73]. Tablets with diazepam and PVA were produced by Obeid et al. In this research, the primary objective was to harness the potential of artificial neural networks, a form of deep learning technology, to generate a predictive model for the release of the API. Various tablet shapes were designed. Factors such as infill density, infill pattern, and surface-to-volume ratio were analyzed. Through the application of Self-Organizing Maps (SOMs) analysis, the researchers concluded that the zigzag infill pattern resulted in a faster release of the drug from the tablets [74]. A Texture analyzer setup was developed for the simulation of the printing process. Using feed force data from different formulations, Gottschalk et al. established a feed force limit for the 3D printer. This allowed them to identify a reliable printing range and ensure reproducibility and accurate target mass. Consequently, the printing process optimization was possible, including speed, time, and temperature, for various materials and formulations. Tablets with ketoconazole in 20% and 40% concentration and Parteck MXP^®^ were 3D-printed in this study [75]. A notion that encodes binary digits on the surface of 3DP geometries containing praziquantel or pramipexole and Parteck MXP^®^, blind watermarking, was investigated by Windolf et al. Colorants played a role in enhancing traceability by making it possible to visually distinguish between different batches or formulations. An API addition was also helpful in the detection process. This notion was presented as ground-breaking, providing a solution for traceability concerns associated with 3D-printed dosage forms [16].

#### 5.2.2. Sustained Release Tablets

Because sustained release tablets have an important role in the present pharmaceutical industry, many studies have concentrated on 3D printing of this type of tablet to perceive their suitability. Tablets with more than one compartment were produced or with different geometries to modulate the drug’s release pattern.

Gioumouxouzis et al. created a three-compartment dosage form with a prolonged release, with the upper and bottom layers being inert polylactic acid caps and the middle layer being a hydrochlorothiazide-loaded Mowiol^®^ 4-88 PVA-mannitol mix. The development of hydrophilic matrices with zero order kinetics has been a highly researched subject, but this was the first study to use FDM technology for the production of solid dosage forms with these characteristics using custom-made filament [76]. In another study, Tagami et al. used polymer fillers such as PVA and polylactic acid (PLA) to modulate calcein’s release. Drug-PVA/PVA and drug-PVA/PLA composite tablets were created and showed acceptable formability. The use of the polymer filler component was successful in modulating calcein release. The PLA-containing tablets had zero-order release kinetics. The PVA-filler tablets were produced by varying the filler’s thickness, resulting in various medication release lag time frames [77]. In a study by Windolf et al., a dose-independent drug release could be achieved using a hollow cylinder-based geometry model designed to make dose adjustments possible without influencing the API’s release profile. This model enabled the dose to be changed in small portions, individually responding to patient demands while utilizing only one filament. Parteck MXP^®^ was associated with mannitol and fumed silica as filament-forming polymer in a praziquantel formulation [14]. Tablets with a controlled release mediated by erosion, consisting of paracetamol and PVA in the form of commercially available filament, cut and then mixed with the API, were manufactured by Goyanes et al. These tablets had different geometries: pyramid, sphere, torus, cylinder, and cuboid. The work made the initial suggestion that 3DP provides a manufacturing path for dosage forms with unique geometries that were not before achievable. Geometry could modify drug dissolving profiles. The resulting drug release rates were as follows, starting with the fastest release: pyramid, torus, cube, sphere, and cylinder [78]. The model active substance felodipine was efficiently formulated as solid dispersions utilizing FDM 3D printing and polymer mixtures of PEG, PEO, and Tween 80 with either Eudragit E PO or Soluplus, by Alhijjaj et al. A PVA-based solid dispersion was employed as a comparison standard for the processability of the polymer mix systems. The outcomes showed how the drug release behavior of the dispersions was influenced by the complex interactions between the compatibility of the excipients in the blends, the polymer’s solubility in the medium, and the creation of interfaces between printed strips during the FDM printing [56]. A study by Saviano et al. involved preparing various solid mixtures using PVA batches with five different particle sizes, ranging from 250 μm to 4000–5000 μm, and ciprofloxacin hydrochloride as API. Finer PVA batches (250–600 µm) resulted in better processability, more efficient drug loading and homogeneity, and reduced drug loss during mixing and extrusion [79]. Obeid et al. also created modified release tablets, containing amlodipine, Parteck^®^ as the polymeric carrier and superdisintegrants to optimize the release. This research explored how different infill patterns in 3D-printed tablets, combined with excipients and varied wall thickness, affect drug release. The tablets were printed in various patterns (zigzag, cubic, tri-hexagon, concentric) with a consistent infill density of 20%. The study found that the zigzag pattern and sodium starch glycolate excipient led to 40% amlodipine release in only 10 min, the fastest drug release, while HPMC HME 4 M (hydroxypropyl methylcellulose) delayed it, for example, 40% amlodipine was released in 200 min [80]. The study conducted by Matijašić, et al. focused on developing filaments with dronedarone hydrochloride, a medication used to treat heart arrhythmias. Different combinations of API, polyethylene glycol (PEG), and PVA filament were examined. The filaments and 3D-printed tablets were analyzed to confirm drug stability and content preservation during processing. The study determined that a blend with 10% PEG, 10% API, and 80% PVA was the most suitable for extrusion and tablet printing, ensuring consistent drug release over 24 h [81]. By developing a co-crystal with nicotinamide, the melting point of hydrochlorothiazide was altered. Consequently, modified release tablets containing Parteck^®^ MXP as a filament-forming polymer and sorbitol as plasticizer were manufactured by Kozakiewicz-lata et al. According to the authors, other high melting point compounds could be successfully processed using the suggested method [82]. Qijun et al. produced DuoTablets, controlled release drug delivery devices, containing 2.2% and 4.8% glipizide and a commercially available PVA that was sheared, milled, and then mixed with the drug for the preparation of filaments. The release pattern followed Korsmeyer–Peppas kinetics. The device consisted of two layers, and the authors reported that the external layer’s characteristics influenced the internal compartment’s release profile. The cylindrical shape was chosen because of its ease of printing, and drug release was fine-tuned by adjusting the drug ratios in layers, with glipizide in the external layer releasing first [83].

In order to make co-administration and once-daily dosage of the two regimens possible, a two-compartment anti-diabetic formulation combining metformin with sustained release and glimepiride with immediate release was created, using Mowiol^®^ 4-88 for the immediate release glimepiride-loaded layer and Eudragit^®^ RL to achieve prolonged release for the metformin-loaded layer. The flat, cylindrical, smooth-edged design was chosen as the favored one. Because of the higher pressure created by the large screw of the single-screw extruder, this type of extruder generated more compact filaments [15]. Vervaet et al. tested filaments with various paracetamol concentrations (0–50% *w*/*w*) and storage conditions (20–40 °C temperature, 11–75% humidity) to evaluate their suitability for 3D printing. The research focused on monitoring polymorphic transitions, as well as evaluating PVA’s role as a drug carrier and stabilizer. Dissolution tests revealed consistent paracetamol release from printed tablets regardless of filament storage time. Higher paracetamol content decreased the extrusion temperature but resulted in rougher filaments due to incomplete API dissolution in PVA. Increased paracetamol content additionally made the filaments brittle, affecting their printing process. They found that the optimal storage conditions were 11% relative humidity, allowing PVA to maintain its interactions with the API and stabilize amorphous paracetamol for at least 12 weeks [84]. The impact of disintegrants was of major interest to Đuranovic et al. They prepared tablets with different release profiles containing paracetamol as API, Parteck^®^ MXP as filament-forming polymer and different disintegrants, which were evaluated for their effect. Distinct strategies were used to enhance drug release, such as adding enhancers, adjusting infill density, and increasing enhancer concentrations. The most successful approach involved using Kollidon CL and mannitol, resulting in tablets reaching a plateau within 2 h, although not meeting the immediate release criteria. Physiologically based biopharmaceutical modeling supported these findings. This was the first study using this type of modeling [85].

#### 5.2.3. Special Designs

The combination of HME and FDM can be used for the creation of personalized dosage forms customized according to individual patient needs. This can be beneficial, especially in tailoring drug doses for specific patient populations or addressing unique therapeutic requirements, like designs appealing to the pediatric population to increase their adherence to medication or designs with qualities that discourage abuse with a high mechanical resistance.

Among the special designs developed through FDM 3DP, capsule-shaped tablets or caplets have been proposed by several authors. Thus, caplets with paracetamol and commercially available PVA filament were developed to evaluate how the internal structure, loading, and content of the drug influence its dissolution. The various drug release characteristics could not be explained by examining the caplets’ porosity [86]. To prove that the combination of HME and FDM 3DP serves as a platform for the creation of individually tailored opioid dose forms with abuse-deterrent qualities, immediate release egg-shaped tablets were developed by Nukala et al. with Parteck^®^ MXP, sorbitol as plasticizer, and metformin hydrochloride as API, in 5, 10, and 15% concentrations. According to FDA recommendations, the egglets were assessed for mechanical manipulation using common household tools, milling, particle size propagation, extraction of solvents, and active substance release. The tablets passed most of the evaluations outlined [87]. Modified release caplets containing budesonide and PVA were developed by Goyanes et al. and examined in comparison with the commercial products Cortiment^®^ and Entocort^®^. The 3D-printed caplets were coated with Eudragit L100 to create a gastro-resistant dosage form. The coated 3D-printed object was resistant to acidic environments and released the API in the small intestinal segment after around an hour. The new product’s release properties could make it potentially useful in treating inflammatory bowel illness [88]. An additional study by Goyanes et al. focused on the creation of modular capsules. A PVA concentrically compartmental Can-capsule, useful for delivering the APIs to the small intestine, and a modular Super-H capsule with various membrane thicknesses were developed. Printed capsules were filled with powdered dronedarone hydrochloride and ascorbic acid [93]. Palekar et al. developed modified release minicaplets with baclofen, Parteck^®^ MXP, and sorbitol for pediatric use. PVA with 10% sorbitol was chosen to manufacture baclofen-loaded filaments based on the findings of the three-point bend test and on the possibility of a wide processing window. Compared to linear, sharkfill, and hexagonal patterns, minicaplets printed in a diamond infill pattern with 100% infill demonstrated a longer disintegration time [61].

The fabrication of orodispersible dosage forms through 3D printing was also explored in several publications. Kurek et al. prepared filaments with a high concentration of fluconazole to manufacture orodispersible immediate release 3D-printed tablets. This high API content, 70% in a printable filament, is a unique aspect. The researchers confirmed the 12-month stability of the formulation. Storage conditions did not affect the quality of the tablets, and all printed tablets released over 95% of fluconazole within 30 min. The tablets’ mechanical properties decreased with higher fluconazole content, but their disintegration patterns remained consistent. The printed tablets exhibited thermodynamic stability for two weeks, ensuring consistency [12]. The same research group produced orodispersible films with aripiprazole and PVA 4-88. Physicochemical and mechanical properties were compared between the 3D-printed and the cast films. Aripiprazole amorphization and the porous structure of printed films enhanced dissolution rates compared to cast ones, despite the latter having better mechanical properties. Extruding PVA with aripiprazole proved challenging due to differences in polymer and API particle morphology. Ensuring a uniform mixture by wetting the PVA flakes with ethanol and then mixing with aripiprazole resolved this problem. Data variations for mass and thickness uniformity were lower in 3D-printed films than in cast ones [90].

Taking into account that design modulation can influence the gastroretention of the dosage forms, the 3DP technology has been indicated as suitable for the design of gastroretentive dosage forms. Chen et al. explored the feasibility of creating gastric floating tablets using FDM 3D printing technology. Ellipsoid-shaped tablets with low infill percentages and propranolol concentrations of 15% and 25% were printed. Glycerol was used as a plasticizer to enhance the properties of the filaments. Both 15% and 25% propranolol tablets floated with no delay and released the drug in a gradual manner. The tablet containing 25% API showed better characteristics, including less weight variation, higher hardness, shorter floating time, and longer drug release [91]. Windolf et al. created the first floatable polypill with prolonged release consisting of pramipexole, levodopa, and benserazide. The floating mini-polypill was created to help patients who have trouble swallowing and allow for customized doses with an extended API release. Its use would make the dosage adjustment easier in response to ON–OFF occurrences. [92].

### 5.3. Other Applications of PVA in 3D Printing

In addition to using PVA in extruding API-containing filaments that are further printed into oral dosage forms via FDM, some studies have utilized PVA for other purposes. The following studies covered the use of PVA in a variety of processes, including the design of dosage forms using powdered, liquid, or injectable APIs. Additionally, PVA was used in the FDM-based passive diffusion printlet creation process. Moreover, other research looked into the stability and into unique qualities that might have a big impact on the final printlet’s characteristics. Furthermore, by combining HME and FDM with other methods, researchers aimed to deepen their grasp on this area.

One of these studies designed a 3D-printed bentobox model enclosing propranolol hydrochloride powder inside its chambers to promote the controlled release. The 3D-printed bentoboxes’ physicochemical characteristics were studied according to the varied infill percentage [94]. Another group of researchers prepared capsule shells containing PVA to be then filled with a solid or liquid API-consisting carrier. Similar to the study mentioned above, this technique was used to regulate the induction time of a delayed release for regional absorption, using a varying wall thickness [95]. Capsule-shaped floating devices were manufactured to regulate gastric retention and release of a commercial domperidone tablet, Motillium-M^®^. A hollow cap with various wall thicknesses was printed using a hydrophilic, PVA-containing filament to increase the buoyancy of the devices. Hydrophobic polylactic acid filament was used to create the device’s body [96]. Another work offered an understanding of how printing settings, printing speed, and material flow rate affect the production of PVA capsular devices. The dose was chosen in order to compare the capsular device’s effectiveness to that of a marketed hard-gelatin form, which includes 100 mg of sodium cromoglycate [97].

Paracetamol gels were injected PVA scaffolds with various internal architectures, showcasing three geometries: horn, cylinder, and reversed horn. Depending on the model, the tablets had distinct release profiles: the horn tablet had a rapid release, the cylinder dosage form had a constant profile, and the reversed horn had a slow release, which can be used in hypertension therapy [98]. PVA was also employed as a plasticizing agent to fine-tune the release of ibuprofen from tablets containing ethyl cellulose as the main excipient. Drug release could be slightly improved by the addition of PVA [99].

Other studies used passive diffusion to load active substances into PVA filaments, instead of using HME to prepare the drug-loaded filaments. For instance, metformin hydrochloride round-chanelled tablets were 3D-printed after PVA filaments were immersed in a 10% (*v*/*v*) solution of metformin HCl in ethanol to increase the drug’s solubility [100]. Goyanes et al. 3D-printed tablets containing aminosalicylate with a modified release profile by applying the substances onto commercially available PVA filaments in an ethanolic drug solution [7]. Through incubation in a saturated methanolic solution of prednisolone, the drug was loaded into a PVA filament at roughly 1.9% *w*/*w* in a different study. Using the FDM-based 3D printer, conventional ellipse-shaped solid tablets with extended drug release were created [101]. Floating 3D-printed devices with amoxicillin were compared to an amoxicillin marketed capsule (Sia-Mox^®^) for managing H. Pylori infection. The outcomes demonstrated that the printed device’s float length was much longer than that of the traditional Sia-Mox^®^ capsule [102]. In another study, 3D-printed tablets were made using PVA filaments previously impregnated with 59.01% fluorescein. The impact of printing speed, infill level, and layer thickness on the dissolution rate was examined. Infill percentage was discovered to be the most important factor in determining a higher dissolution rate [103].

Developing methods that mimic the usual storage conditions can be essential for creating safe pharmaceutical dosage forms. A new method for pre-formulation studies for thermal processing and aging of printed drugs assessed their shape, and the thermal, crystallographic, and spectroscopic profiles were studied. It was found that along with physical alterations, the mixture of paracetamol and PVA displayed evidence of thermal instability and chemical degradation [2]. Cerda et al. demonstrated how passive diffusion and FDM can be used to customize the dose of nifedipine. The support vector machine regression is the foundation of the model used for prediction. It would be ideal to use PVA filaments made from low-molecular-weight polymer chains to ensure that 3D-printed tablets dissolve under moderate mixing conditions and temperature. Being pH-independent translates to a speedier and more consistent management of blood pressure. At 25 °C and 60% humidity, the tablets displayed a projected stability of over three years. This shows that 3D-printed medications can be preserved for a very long time, like typically commercially produced solid dosage forms [55].

Employing a drop-on-solid printing approach enables a precise and reproducible operation. Junqueira et al. combined FDM for the production of PVA tablets with inkjet printing for the dispensing of minoxidil ethanolic solutions for a sustained release profile. The drug solution was deposited onto the plain tablet surface using this method, which ejected tiny droplets. Dosage dispensing is quick, and drug doses can be applied with high accuracy and consistency; this combination of methods has resulted in a faster tablet production [104].

Basa et al. focused on evaluating PVA-based carriers for 3D printing. The most popular supporting substance can be improved to become a crucial excipient filament for individualized therapy. Because PVA-based dosage forms break down in the patient’s body in a matter of hours, the phenomena of “ghost tablets” can be eliminated thanks to the polymer’s properties. The CAD design makes it possible to incorporate drug-releasing gaps onto the carrier’s surface [105].

Numerous polymers used regularly in the production of dosage forms were evaluated by Melocchi et al. as materials for HME of filaments compatible with FDM procedures. Eudragit^®^ L, hydroxypropyl methylcellulose acetate succinate, and swellable/erodible (hydrophilic cellulose derivatives, PVA, Soluplus^®^) polymers were successfully processed using a twin-screw extruder, and the ability to use them for creating 600 mm thick disks was shown. The created PVA filaments were, therefore, deemed to be potentially appropriate for printing layers of coating for rapid or modified release, capsules, and dosage forms of any kind when loaded with active substances [62].

A relatively recent area of research is localized medicine delivery. Nanostructured lipid carriers were incorporated into 3D-printed PVA wafers to be used in the treatment of oral cancer. The gradual release from the 3D wafer was seen in a time-dependent way. As the PVA matrix depleted and began to dissolve in synthetic saliva, the release of the entire nanostructured lipid carriers was possible [106].

## 6. Conclusions

To conclude, the field of 3D printing is in continuous development, rising more and more throughout the years. 3DP has the ability to completely transform the processes utilized in the existing pharmaceutical production sector by enabling the creation of personalized dosage forms for every patient in accordance with their particular requirements. The customizable approach enables this technology to address the prevalent problems related to the modulation of drug dosage in the pharmaceutical sector.

PVA is one of the most important filament-forming excipients due to its physical properties, which is why a great number of studies have made use of it to create tablets, films, wafers, capsules, etc., containing APIs to be able to tailor patients’ treatment individually. The research compiled in the article illustrates the potential and growing interest of 3DP in the production of pharmaceuticals. Researchers have investigated a variety of facets of this technology, including printability, drug release profiles, and the influence of numerous factors on tablet characteristics. The trials have shown how flexible 3DP is in designing sophisticated medication delivery devices. It has been successful in manufacturing multilayer polypills, three-compartment solid oral dosage forms, and capsule-shaped devices with controlled drug release. Researchers have also looked into adding APIs or colorants to 3DP tablets in order to provide batch traceability and encode information on their surface. These developments offer hope for safeguarding the legitimacy and standardization of 3DP medicinal goods. Research has also concentrated on improving the ability to dissuade opioid usage and creating modified release formulations for a variety of medications, including anti-inflammatory and anti-diabetic treatments.

Overall, PVA has been, and will remain, a crucial part of 3D printing, from its early days as a support material to the present application of creating filaments to print solid oral dosage forms.

## Figures and Tables

**Table 1:** Pharmaceutical grades of PVA.

Name, Manufacturing Company	Physical Properties	Advantages and Applications	References
Mowiflex^TM^ C 17, Kuraray, Tokyo, Japan Mowiflex^TM^ C 30, Kuraray, Tokyo, Japan	soluble in cold water, at 25 °C melt viscosity of 12 g/10 min processing temperature between 190 and 210 °C for C 17 and 190 and 200 °C for C 30	water-soluble support material for additive manufacturing based on fused filament fabrication (FFF) methods; can yield high filament stiffness; use: to print intricate structures;	[40,41,42,43,44]
KURARAY POVAL^TM^ 4-88, Kuraray, Tokyo, Japan Parteck^®^ MXP 4-88, Merck, Darmstadt, Germany	granules/fine powder fine powder	stabilizing certain compounds with a wide variety of melting temperatures in their amorphous form;	[45,46]
Parteck^®^ MXP 3-82 from Merck, Darnstadt, Germany	crystalline powder	strong interactions with hydrophobic molecules, both in the solid state and in solution; prevents precipitation; can sustain high supersaturation levels; specifically designed for HME.	[46]
GOHSENOL^TM^ EG-03P, Mitsubishi Chemical Performance Polymers (MCPP), Tokyo, Japan	granules	surfactant behavior; minimal skin irritation properties, can be used in cosmetics.	[47]
GOHSENOL^TM^ EG-05P, Mitsubishi Chemical Performance Polymers (MCPP), Tokyo, Japan GOHSENOL^TM^ EG-05PW, Mitsubishi Chemical Performance Polymers (MCPP), Tokyo, Japan	physical form of granules for EG-05P and of powder for EG-05PW; water-soluble;	surfactant behavior; minimal skin irritation properties, can be used in cosmetics.	[48,49]
PVA 0588, Liwei chemical (Sinochem), Guangdong, China	water-soluble	film-forming ability; good stability; chemical and oil resistance; can be combined with rubber, plastics, and water-soluble polymers.	[50]

**Table 2:** Applications of PVA as filament-forming excipient for manufacturing pharmaceutical dosage forms through FDM 3D printing.

Objective	Composition	Extrusion		FDM		References
		Equipment (Name, Provider, Type)	Working Parameters	Equipment	Working Parameters (Build Plate Temperature/Nozzle Temperature/Printing Speed)	
**Immediate release tablets**						
To investigate the effects of lowering processing temperatures by utilizing a temporary plasticizer, water, as well as the impact of polypill design on drug release.	Lisinopril dihydrate, indapamide, amlodipine besylate, rosuvastatin calcium; Parteck MXP; Plasticizer: sorbitol, water;	HAAKE™ MiniCTW, Thermo Scientific™, Karlsruhe, Germany, micro-conical twin-screw compounder	90 °C and 35 rpm	Makerbot 2x, Makerbot, Brooklyn, NY, USA	40 °C/ 150 °C/210 °C/n.d.	[69]
To focus on the processability of PVA.	Carvedilol and Haloperidol; Parteck^®^ MXP; Plasticizer: Parteck^®^ SI 150, sorbitol;	Process 11, Thermo Scientific™, Bridgewater, NJ, USA, corotating twin-screw extruder	Various temperatures and screw speeds, depending on the formulations	Makerbot Replicator 2, MakerBot, Brooklyn, NY, USA	room temperature/ 210 °C/ 50 mm/s	[70]
To develop immediate release tablets under QbD guidance with a high drug loading.	Diclofenac sodium from 10% to 60%; Parteck^®^ MXP;	Noztek Pro, Noztek, Shoreham-By-Sea, UK, single-screw extruder	65 rpm, 190 °C and a 1–2 g/min feeding rate	Makerbot Replicator 2X, Makerbot, Brooklyn, NY, USA	45° C/185–190 °C/30 mm/s	[13]
To compare the fabrication through HME and impregnation.	Indomethacin (30%); Mowiflex^®^ C17 granules from Kuraray, pulverized and commercial PVA filament;	Wellzoom^TM^ C desktop, Shenzhen Mistar Technology Co., Ltd., Shenzen, China, single-screw extruder	180 °C and 12 rpm	Flashforge Creator Pro Printing, Zhejiang Flashforge 3D Technology Co., Ltd., Zhejiang, China	60 °C/200 °C/20 mm/s	[71]
To test formulations based on several polymeric carriers.	Timapripant; Gohsenol^®^ EG 03P; EMDEX^®^, GalenIQ^®^ Tackidex^®^ polyethyleneglycol (PEG) types;	Haake^TM^ MiniLab II, Thermo Scientific™, Madison, US-WI, USA, twin-screw extruder	145 to 180 °C, 40/50 rpm	Kloner3D 240^®^ Twin, Kloner3D, Florence, Italy	50 °C/various temperatures depending on the carriers/25 mm/s	[72]
To reach a novel viewpoint on material concerns when a PVA-based FDM 3D-printed system is the desired outcome.	Ketoprofen (30%); partially hydrolyzed (88%) PVA: Mowiol^®^ 4-88 and Parteck^®^ MXP;	Noztek Pro, Noztek, Shoreham-By-Sea, UK, single-screw extruder	180 °C and 65 rpm	MakerBot Replicator 2X, Makerbot, Brooklyn, NY, USA	80 °C/ 185 °C/ 30 mm/s	[73]
To use deep learning in order to analyze the impact of infill density, infill pattern, and SA/V ratio on medication dissolution rate.	Diazepam (2.5%); 97.5% PVA, from 3D Republic;	Noztek Pro, Noztek, Shoreham-By-Sea, UK, single-screw extruder	205 °C	Ultimaker 3, Ultimaker, Geldermalsen, Netherlands	70 °C/ 185 °C/n.d.	[74]
To determine the feed forces.	Ketoconazole (20% and 40%); Parteck MXP^®^, technical PVA filament.	Pharma 11, Thermo Fisher Scientific™, Waltham, MA, USA, co-rotating twin-screw extruder	zone 1: 80 °C and zone 2: 160 °C, zone 3–7: 210/220 -260/270 °C; 300 rpm	Ultimaker 3, Ultimaker, Geldermalsen, Netherlands	60 °C/220/240 °C/30 mm/s	[75]
Blind watermarking, a notion that encodes binary digits (bits) on the surface of 3D-printed geometries, was investigated.	Praziquantel (5%), pramipexole (5%), and triamcinolone acetonide (5%) in different 3D-printed tablets; Parteck MXP^®^;	Pharmalab HME 16, Thermo Fisher Scientific™, Waltham, MA, USA, co-rotating twin-screw extruder	-	Prusa i3MK3, Prusa Research, Prague, Czech Republic	n.d./differing temperatures depending on the API/n.d.	[16]
**Modified release: sustained release tablets**						
To create a three-compartment solid dosage form via FDM, with an intermediate layer made of a hydrochlorothiazide-loaded PVA-mannito blend and an upper and lower layer made up of inert PLA caps.	10% Hydrochlorotiazide; 84% Mowiol^®^ 4-88; 10% mannitol;	Filabot Original^®^, Filabot, VT, USA, single-screw extruder	170 °C, 35 rpm	MakerBot Replicator 2X, Makerbot, Brooklyn, NY, USA	65 °C/ 200 °C/n.d.	[76]
To modulate medication release with a polymer filler.	Calcein; Gohsenol EG-05P;	n.d., Nippon Synthetic Chemical Industry Co., Ltd.,Osaka, Japan, twin-screw extruder	90/120/180/190/200/200/200/205/210 °C	FDM-200 W, NinjaBot, Shizuoka, Japan	60 °C/ 190 °C/20 mm/s	[77]
To create a model that permits dose adjustments in a tablet without changing the API’s release profile.	Pramipexole, levodopa and praziquantel; Parteck MXP^®^; Mannitol (plasticizer) and silica (glidant).	Pharmalab HME 16, Thermo Fisher Scientific™, Rockford, IL, USA, co-rotating twin-screw extruder	Pramipexole-PVA formulation printing temperatures: 30/100/180/180/180/180/180/195/195 °C; Praziquantel-PVA formulation zones 2–10 temperatures: 21/31/78/180/180/180/180/180/195 °C; Screw speed: 30 rpm.	Prusa i3 Mk3, Prusa Research, Prague, Czech Republic	60 °C/185 °C/20 mm/s	[14]
To create tablets with various geometries, many of which would be difficult to create using powder compaction.	Paracetamol (4%); Commercially available PVA filament, cut and mixed with the API.	FilaBot^®^ hot melt extruder, Filabot, Barre, VT, USA, single-screw extruder.	180 °C and 35 rpm	MakerBot Replicator 2X, Makerbot, Brooklyn, NY, USA	n.d./180 °C/90 mm/s	[78]
To produce felodipine solid dispersions utilizing FDM 3D printing and polymer mixtures of PEG, PEO, and Tween 80 with either Eudragit E PO or Soluplus.	Felodipine (10%); 33–38% partially hydrolyzed PVA; Tween 80 22.5%;	Haake MiniLab II Micro Compounder, Thermo Fisher Scientific™, Karlsruhe, Germany, co-rotating twin-screw extruder	130 °C and screw speed 100 rpm, decreasing to 25 rpm through the process	MakerBot Replicator 2, Makerbot, Brooklyn, NY, USA	Room temperature/ 150 °C/n.d.	[56]
To study and demonstrate the significance of PVA particle size for the extrusion and printing processes, in addition to drug adhesion to the polymer.	Ciprofloxacin hydrochloride; Commercially available PVA, five PVA samples (4000–5000 μm, 1000–2000 μm, 600–1000 μm, 250–600 μm, 250 μm).	Noztek Touch HT, Noztek,, Shoreham-By-Sea, UK, single-screw extruder	T1 varied in accordance with the PVA size; T2 at 175 °C; 30 to 60 rpm	Ultimaker 3, Ultimaker, Geldermalsen, Netherlands	80 °C/195 °C/8 mm/s	[79]
To emphasize the significance of modifying printer settings and using excipients to create tailored-release medication.	Amlodipine; Parteck^®^; Sodium starch glycolate and hydroxypropyl methylcellulose, HPMC HME 4 M HYDROMELLOSE;	Noztek Pro, Noztek, Shoreham-By-Sea, England, single-screw extruder	150–160 °C	Ultimaker 3, Ultimaker, Geldermalsen, Netherlands	80 °C/180–190 °C/n.d.	[80]
To create filaments for 3D printing by choosing the right processing parameters and creating polymer blends with the suitable composition.	Powder mixture: 10% dronedarone powder, 80% of small-diameter PVA filament and 10% PEG in powder form, plasticizer;	Noztek Pro, Noztek, Shoreham-By-Sea, UK, single-screw extruder	170 °C, 2.5 m/min	Flashforge Inventor I, Zhejiang Flashforge 3D Technology Co., Ltd., Zhejiang, China	40 °C/200 °C/10 mm/s	[81]
To assess the impact of the drug phase and its miscibility with polymer/plasticizer mixtures on the mechanical characteristics of generated filaments.	Hydrochlorothiazide; nicotinamide to obtain a co-crystal; Parteck^®^ MXP; Plasticizer: Sorbitol (Parteck^®^ SI 150) 10%, 20%, and 30%;	Process 11, Thermo Fisher Scientific™, Karlsruhe, Germany, co-rotating twin-screw extruder	70 to 170 °C, 120 rpm	ME Builder Premium Small 3D device, Builder, Noordwijkerhout, Netherlands	n.d./190 °C/40 mm/s	[82]
To use modeling software in order to create oral drug delivery systems with intricate internal structures.	Glipizide, 2.2 and 4.8%; Commercially available PVA filament, sheared, milled and mixed with the drug;	LSJ20, Shanghai Kechuang Ltd., Shanghai, China, single-screw extruder	180 °C, 15 rpm	Clouovo Delta-MK2, Clouovo Technologies Inc., Shenyang, China	n.d./195 °C/15 mm/s	[83]
**Modified release tablets, multiple APIs**						
To create a two-compartment anti-diabetic formulation for co-administration in one dosage form, once a day.	Glimepiride and metformin; Mowiol^®^ 4-88 for the immediate-release glimepiride-loaded layer; Eudragit^®^ RL: prolonged release for the metformin-loaded layer; Plasticizers: mannitol/citric acid monohydrate/PEG 400/triethyl citrate;	Filabot Original^®^ single-screw Extruder, Filabot Inc., VT, USA, single-screw extruder & HAAKE MiniLab^®^ extruder, Thermo Scientific, MA, USA, co-rotating twin-screw extruder	190 °C, 23 rpm	MakerBot Replicator^®^ 2, Makerbot, Brooklyn, NY, USA	90 °C/205 °C/70 mm/s	[15]
**Immediate and modified release tablets**						
To assess how easily paracetamol-containing PVA-based filaments could be processed by preparing tablets with slow release–more PVA, and with immediate release–more paracetamol.	Paracetamol; EMPROVE^®^ poly(vinyl) alcohol 4-88;	Prism Eurolab 16, Thermo Fisher, Karlsruhe, Germany, co-rotating twin-screw extruder	100 rpm	MakerBot Replicator 2, Makerbot, Brooklyn, NY, USA	n.d./different temperatures depending on the formulation/ 90 mm per minute	[84]
To investigate the impact on the drug release using four alternative approaches with release boosters.	Paracetamol (30–40% *w*/*w*); Parteck^®^ MXP (45–55% *w*/*w*); Affinisol^TM^ HPMC HME 4 M (5% *w*/*w*), Primellose^®^, Kollidon CL, PARTECK^®^ M 200, EXPLOTAB^®^.	Noztek Pro, Noztek, Shoreham-By-Sea, UK, single-screw extruder	120 °C	Ultimaker 3, Ultimaker, Geldermalsen, Netherlands	70 °C/150 °C/35 mm/s	[85]
**Special designs**						
To prepare capsule-shaped tablets and assess how drug loading, content, and internal structure (micropore volume) affect drug dissolution characteristics.	Paracetamol (4.3 and 8.2%) and caffeine (4.7 and 9.5%); commercially available PVA filament from Makerbot.	Noztek Pro, Noztek, Shoreham-By-Sea, UK, single-screw extruder	180 °C, 15 rpm	MakerBot Replicator 2X, Makerbot, Brooklyn, NY, USA	n.d./200 °C/90 mm/s	[86]
To prove that the combination of HME and FDM provides a platform for the creation of abuse deterrent patient-tailored opioid immediate release egg-shaped dosage forms.	Metformin hydrochloride (5; 10; 15%); Parteck^®^ MXP; Plasticizer: sorbitol.	Process 11, Thermo Fisher Scientific™, Waltham, MA, USA, parallel twin-screw extruder	Placebo filament: 170 °C; Metformin-loaded filament: 170 °C and 100 rpm.	MakerBot^®^ Replicator 2, Makerbot, Brooklyn, NY, USA	Room temperature/200 °C/45 mm/s	[87]
To combine HME with FDM and film coating in order to create modified release caplets.	Budesonide; PVA commercial filaments from Makerbot Inc.; Coating polymer: Eudragit^®^ L100;	Noztek Pro, Noztek, Shoreham-By-Sea, UK, single-screw extruder	170 °C, 15 rpm	MakerBot Replicator 2X, Makerbot, Brooklyn, NY, USA	n.d./190 °C/90 mm/s	[88]
To create solid capsule-shaped devices with two layers, DuoCaplets, one with immediate release and one with delayed release.	Paracetamol (4.3/8.2%), caffeine (4.7/9.5%); Commercially available PVA filament (milled and sieved).	Noztek Pro, Noztek, Shoreham-By-Sea, UK, single-screw extruder	180 °C, 15 rpm	MakerBot Replicator 2X, Makerbot, Brooklyn, NY, USA	n.d./200 °C/90 mm/s	[89]
To investigate the use of 3D printing to create customized baclofen modified release minicaplets for the pediatric market.	Baclofen; Parteck^®^ MXP; Plasticizer: Sorbitol (Parteck^®^ SI 150);	Process 11, Thermo Fisher Scientific™, Waltham, Massachusetts, USA, parallel twin-screw extruder	160 °C, 200 rpm	MakerBot, Makerbot, Brooklyn, NY, USA	n.d./190 °C/40 mm/s	[61]
To produce an FDM-printable filament with a high API concentration and then print it in the form of orodispersible tablets.	Fluconazole (10% to 70%); Parteck^®^ MXP;	RES-2P/12A Explorer, Zamak Mercator^®^, Skawina, Poland., corotating twin-screw extruder	Varying temperatures depending on fluconazole concentration	ZMorph^®^ 2.0S, Zmorph^®^, Wroclaw, Poland	n.d./n.d./10 mm/s	[12]
To test and compare the mechanical and physicochemical characteristics of 3D-printed and casted orodispersible films.	Aripiprazole; Poval 4-88 from Kuraray, PVA commercially available filament.	Noztek1 Pro, Noztek, Shoreham-By-Sea, UK, single-screw extruder	172 °C	Zmorph1 2.0S, Zmorph^®^, Wroclaw, Poland	Control films: n.d./185 °C/10 mm/s; API-containing films: n.d./190 °C/5 mm/s	[90]
To assess the effect of the infill percentage on the gastric floating tablets’ qualities.	10% Propranolol hydrochloride; PVA 0588; Plasticizer: glycerol;	FilaBot^®^ FOV1, Filabot, Barre, VT, USA, single-screw hot melt extruder	142 °C, 35 rpm	MakerBot Replicator 2X desktop 3D printer, Makerbot, Brooklyn, NY, USA	111 °C/185 °C/10–15 mm/s	[91]
To create the first printed oral dosage form including pramipexole, levodopa, and benserazide in the form of a prolonged release floatable polypill.	Levodopa/benserazide (4:1) (extended drug release), pramipexole (with PVA for quick drug release); Parteck MXP; Plasticizer: 10% mannitol	Pharmalab, Thermo Fisher Scientific™, Waltham, MA, USA, co-rotating twin-screw extruder	20/20/100/180/180/180/180/195/195 °C	Prusa 3D printer, Prusa Research, Prague, Czech Republic	70 °C/185 °C/n.d.	[92]

## References

[1] Trenfield S.J., Madla C.M., Basit A.W., Goyanes A. (2021). The Shape of Things to Come: Emerging Applications of 3D Printing in Healthcare Translating 3D printed pharmaceuticals: From hype to real-world clinical applications. Adv. Drug Deliv. Rev..

[2] Silva I.A., Luiza A., Gratieri T., Gelfuso G.M., Sa-barreto L.L., Cunha-filho M. (2022). Compatibility and stability studies involving polymers used in fused deposition modeling 3D printing of medicines. J. Pharm. Anal..

[3] Pitzanti G., Mathew E., Andrews G.P., Jones D.S., Lamprou D.A. (2022). 3D Printing: An appealing technology for the manufacturing of solid oral dosage forms. J. Pharm. Pharmacol..

[4] Parulski C., Jennotte O., Lechanteur A., Evrard B. (2021). Challenges of fused deposition modeling 3D printing in pharmaceutical applications: Where are we now?. Adv. Drug Deliv. Rev..

[5] Nasereddin J.M., Wellner N., Alhijjaj M., Belton P., Qi S. (2018). Development of a Simple Mechanical Screening method for predicting the feedability of a pharmaceutical FDM 3D printing filament. Pharm. Res..

[6] Patel N.G., Serajuddin A.T.M. (2023). Improving drug release rate, drug-polymer miscibility, printability and processability of FDM 3D-printed tablets by weak acid-base interaction. Int. J. Pharm..

[7] Goyanes A., Buanz A.B.M., Hatton G.B., Gaisford S., Basit A.W. (2015). 3D printing of modified-release aminosalicylate (4-ASA and 5-ASA) tablets. Eur. J. Pharm. Biopharm..

[8] Iftekar S.F., Aabid A., Amir A., Baig M. (2023). Advancements and Limitations in 3D Printing Materials and Technologies: A Critical Review. Polymers.

[9] Satoh K. (2014). Poly(vinyl alcohol) (PVA). Encyclopedia of Polymeric Nanomaterials.

[10] Asthana N., Pal K., Aljabali A.A.A., Tambuwala M.M., de Souza F.G., Pandey K. (2021). Polyvinyl alcohol (PVA) mixed green–clay and aloe vera based polymeric membrane optimization: Peel-off mask formulation for skin care cosmeceuticals in green nanotechnology. J. Mol. Struct..

[11] Rowe R.C., Sheskey P.J., Quinn M.E., Rowe R.C., Sheskey P.J., Quinn M.E. (2009). Handbook of Pharmaceutical Expicients.

[12] Kurek M., Urszula B., Loskot J., Kramarczyk D., Paluch M., Jachowicz R. (2023). Preparation and advanced characterization of highly drug-loaded, 3D printed orodispersible tablets containing fluconazole. Int. J. Pharm..

[13] Crișan A.G., Iurian S., Porfire A., Rus L.M., Bogdan C., Casian T., Lucacel R.C., Turza A., Porav S., Tomuță I. (2021). QbD guided development of immediate release FDM-3D printed tablets with customizable API doses. Int. J. Pharm..

[14] Windolf H., Chamberlain R., Quodbach J. (2022). Dose-independent drug release from 3D printed oral medicines for patient-specific dosing to improve therapy safety. Int. J. Pharm..

[15] Gioumouxouzis C.I., Baklavaridis A., Katsamenis O.L., Markopoulou C.K., Bouropoulos N., Tzetzis D., Fatouros D.G. (2018). A 3D printed bilayer oral solid dosage form combining metformin for prolonged and glimepiride for immediate drug delivery. Eur. J. Pharm. Sci..

[16] Windolf H., Chamberlain R., Delmotte A., Quodbach J. (2022). Blind-Watermarking—Proof-of-Concept of a Novel Approach to Ensure Batch Traceability for 3D Printed Tablets. Pharmaceutics.

[17] Nthoiwa K.K.M., Diaz C.A., Chaudhari Y. (2016). Vinyl alcohol polymers. Handb. Thermoplast. Second Ed..

[18] Muppalaneni S. (2013). Polyvinyl Alcohol in Medicine and Pharmacy: A Perspective. J. Dev. Drugs.

[19] Owen K. (1955). Polyvinyl Alcohol Sponge as an Arterial Substitute. Proc. R. Soc. Med..

[20] Baker M.I., Walsh S.P., Schwartz Z., Boyan B.D. (2012). A review of polyvinyl alcohol and its uses in cartilage and orthopedic applications. J. Biomed. Mater. Res. B. Appl. Biomater..

[21] Nilforoushzadeh M.A., Amirkhani M.A., Zarrintaj P., Moghaddam A.S., Mehrabi T., Alavi S., Mollapour M. (2018). Skin care and rejuvenation by cosmeceutical facial mask. J. Cosmet. Dermatol..

[22] Patel A.R., Vavia P.R. (2010). Evaluation of Synthesized Cross Linked Polyvinyl Alcohol as Potential Disintegrant. J. Pharm. Pharm. Sci..

[23] Morita R., Honda R., Takahashi Y. (2000). Development of oral controlled release preparations, a PVA swelling controlled release system (SCRS). II. In vitro and in vivo evaluation. J. Control Release.

[24] Swarbrick J. (2019). Encyclopedia of Pharmaceutical Technology VOLUME 1.

[25] Umemoto Y., Uchida S., Yoshida T., Shimada K., Kojima H., Takagi A., Tanaka S., Kashiwagura Y., Namiki N. (2020). An effective polyvinyl alcohol for the solubilization of poorly water-soluble drugs in solid dispersion formulations. J. Drug Deliv. Sci. Technol..

[26] Krishna N., Brow F. (1964). Polyvinyl alcohol as an ophthalmic vehicle. Effect on regeneration of corneal epithelium. Am. J. Ophthalmol..

[27] Saettone M.F., Giannaccini B., Chetoni P., GALLI G., Chiellini E. (1984). Vehicle effects in ophthalmic bioavailability: An evaluation of polymeric inserts containing pilocarpine. J. Pharm. Pharmacol..

[28] Falavarjani K.G. (2009). Implantable posterior segment drug delivery devices; novel alternatives to currently available treatments. J. Ophthalmic Vis. Res..

[29] Takeuchi H., Kojima H., Yamamoto H., Kawashima Y. (2000). Polymer coating of liposomes with a modified polyvinyl alcohol and their systemic circulation and RES uptake in rats. J. Control Release.

[30] Gajra B., Pandya S.S., Vidyasagar G., Rabari H., Dedania R.R., Rao S. (2012). Poly vinyl alcohol hydrogel and its pharmaceutical and biomedical applications: A review. Int. J. Pharm. Res..

[31] Ultimaker PVA Technical Data Sheet. 1–2. https://support.ultimaker.com/s/article/1667411286876.

[32] Bianchi M., Pegoretti A., Fredi G. (2023). An overview of poly(vinyl alcohol) and poly(vinyl pyrrolidone) in pharmaceutical additive manufacturing. J. Vinyl Addit. Technol..

[33] Paul J.S., Walter G.C., Colin G.C. (2020). Handbook of Pharmaceutical Excipients. Remington: The Science and Practice of Pharmacy.

[34] Saxena S.K. (2004). Polyvinyl alcohol (PVA) chemical and technical assessment (CTA) first draft prepared by. Chem. Tech. Assess.

[35] Fong R.J., Robertson A., Mallon P.E., Thompson R.L. (2018). The impact of plasticizer and degree of hydrolysis on free volume of poly(vinyl alcohol) films. Polymers.

[36] Nagarkar R., Patel J. (2019). Polyvinyl Alcohol: A Comprehensive Study. ACTA Sci. Pharm. Sci..

[37] Vinyl Alcohol|CH2CHOH-PubChem. https://pubchem.ncbi.nlm.nih.gov/compound/11199#section=Other-Experimental-Properties.

[38] Babaie A., Madadkhani S., Stoeber B. (2014). Evaporation-driven low Reynolds number vortices in a cavity. Phys. Fluids.

[39] Kasselkus A., Weiskircher-Hildebrandt E., Schornick E., Bauer F., Zheng M. (2018). Polyvinyl alcohol: Revival of a long lost polymer. Pharma BioPharma Raw Mater. Solut..

[40] Kuraray Mowiflex 3D Printing Guidelines. https://www.kuraray-poval.com/fileadmin/technical_information/brochures/mowiflex/mowiflex_3d_printing_guidelines.pdf.

[41] Kuraray (2023). MOWIFLEX TM C 17 Technical Data Sheet MOWIFLEXTM C 17 Properties MOWIFLEXTM C 17 Processing Guidelines Extrusion. https://www.kuraray-poval.com/fileadmin/user_upload/KURARAY_POVAL/technical_information/grades_by_region/grades_mowiflex2/TDS-Mowiflex-C17.pdf.

[42] Kuraray (2021). MOWIFLEX TM C 30 Technical Data Sheet MOWIFLEX TM C 30 Properties MOWIFLEX TM C 30 Processing Guidelines. https://www.kuraray-poval.com/fileadmin/user_upload/KURARAY_POVAL/technical_information/grades_by_region/grades_mowiflex2/TDS-Mowiflex-C30.pdf.

[43] Kuraray America, Kuraray Europe MOWIFLEXTM Combining Strengths of Thermoplastics and PVOH. https://www.kuraray-poval.com/fileadmin/technical_information/brochures/mowiflex/mowiflex_general_information_combining_strengths_of_thermoplastics_and_pvoh.pdf.

[44] Kuraray Poval Mowiflex TM as a Water Soluble Support Material for Additive Manufacturing Your Products. https://www.kuraray-poval.com/fileadmin/technical_information/brochures/mowiflex/mowiflex_water_soluble_support_material_for_3d_printing.pdf.

[45] Kuraray (2022). KURARAY POVAL TM 4-88 Technical Data Sheet KURARAY POVAL TM 4-88 Properties. https://www.knowde.com/stores/kuraray/products/kuraray-kuraray-poval-4-88.

[46] Parteck® MXP Excipient for Hot Melt Extrusion|Small Molecule Pharmaceuticals|Merck. https://www.merckmillipore.com/BE/fr/products/small-molecule-pharmaceuticals/formulation/solid-dosage-form/parteck-excipients/parteck-mxp/Ieyb.qB.lAcAAAFYLEQeWww_,nav?ReferrerURL=https%3A%2F%2Fwww.google.com%2F&bd=1.

[47] Mitsubishi Chemical Performance Polymers (MCPP) (2022). GOHSENOL TM EG-03P Technical Data Sheet GOHSENOL TM EG-03P Properties. https://omnexus.specialchem.com/product/t-mitsubishi-chemical-performance-polymers-mcpp-gohsenol-eg-03p.

[48] Mitsubishi Chemical Performance Polymers (MCPP) (2022). GOHSENOL TM EG-05P Technical Data Sheet GOHSENOL TM EG-05P Properties. https://omnexus.specialchem.com/product/t-mitsubishi-chemical-performance-polymers-mcpp-gohsenol-eg-05p.

[49] Mitsubishi Chemical Performance Polymers (MCPP) (2022). GOHSENOL TM EG-05PW Technical Data Sheet GOHSENOL TM EG-05PW Properties. https://omnexus.specialchem.com/product/t-mitsubishi-chemical-performance-polymers-mcpp-gohsenol-eg-05pw.

[50] Liwei Chemical (Sinochem) (2022). PVA 0588 Technical Data Sheet PVA 0588 Properties. https://omnexus.specialchem.com/product/t-liwei-chemical-sinochem-pva-0588.

[51] BASF (2015). Kollicoat ® IR ® Technical Information. https://pharma.basf.com/technicalinformation/30132288/kollicoat-ir.

[52] Colorcon (2023). OPADRY® II Product Information. https://www.colorcon.com/markets/pharmaceuticals/film-coatings/immediate-release/opadry-ii.

[53] Okafor-muo O.L., Hassanin H., Kayyali R., Elshaer A. (2020). 3D Printing of Solid Oral Dosage Forms: Numerous Challenges With Unique Opportunities. J. Pharm. Sci..

[54] Durga Prasad Reddy R., Sharma V. (2020). Additive manufacturing in drug delivery applications: A review. Int. J. Pharm..

[55] Cerda J.R., Arifi T., Ayyoubi S., Knief P., Paloma Ballesteros M., Keeble W., Barbu E., Marie Healy A., Lalatsa A., Serrano D.R. (2020). Personalised 3d printed medicines: Optimising material properties for successful passive diffusion loading of filaments for fused deposition modelling of solid dosage forms. Pharmaceutics.

[56] Alhijjaj M., Belton P., Qi S. (2016). An investigation into the use of polymer blends to improve the printability of and regulate drug release from pharmaceutical solid dispersions prepared via fused deposition modeling (FDM) 3D printing. Eur. J. Pharm. Biopharm..

[57] Fuenmayor E., Forde M., Healy A.V., Devine D.M., Lyons J.G., McConville C., Major I. (2018). Material considerations for fused-filament fabrication of solid dosage forms. Pharmaceutics.

[58] Boetker J., Water J.J., Aho J., Arnfast L., Bohr A., Rantanen J. (2016). Modifying release characteristics from 3D printed drug-eluting products. Eur. J. Pharm. Sci..

[59] Kramarczyk D., Knapik-Kowalczuk J., Kurek M., Jamróz W., Jachowicz R., Paluch M. (2023). Hot Melt Extruded Posaconazole-Based Amorphous Solid Dispersions—The Effect of Different Types of Polymers. Pharmaceutics.

[60] Digkas T., Porfire A., Van Renterghem J., Samaro A., Borodi G., Vervaet C., Crișan A.G., Iurian S., De Beer T., Tomuta I. (2023). Development of Diclofenac Sodium 3D Printed Cylindrical and Tubular-Shaped Tablets through Hot Melt Extrusion and Fused Deposition Modelling Techniques. Pharmaceuticals.

[61] Palekar S., Kumar P., Mishra S.M., Kipping T., Patel K. (2019). Application of 3D printing technology and quality by design approach for development of age-appropriate pediatric formulation of baclofen. Int. J. Pharm..

[62] Melocchi A., Parietti F., Maroni A., Foppoli A., Gazzaniga A., Zema L. (2016). Hot-melt extruded filaments based on pharmaceutical grade polymers for 3D printing by fused deposition modeling. Int. J. Pharm..

[63] Ilyés K., Kovács N.K., Balogh A., Borbás E., Farkas B., Casian T., Marosi G., Tomuță I., Nagy Z.K. (2019). The applicability of pharmaceutical polymeric blends for the fused deposition modelling (FDM) 3D technique: Material considerations–printability–process modulation, with consecutive effects on in vitro release, stability and degradation. Eur. J. Pharm. Sci..

[64] Pinho L.A.G., Luiza A., Livia L., Barreto L.S., Gratieri T., Gelfuso G.M. (2021). Preformulation Studies to Guide the Production of Medicines by Fused Deposition Modeling 3D Printing. PharmSciTech.

[65] Elbadawi M. (2019). Rheological and Mechanical Investigation into the Effect of Different Molecular Weight Poly(ethylene glycol)s on Polycaprolactone-Ciprofloxacin Filaments. ACS Omega.

[66] Aho J., Bøtker J.P., Genina N., Edinger M., Arnfast L., Rantanen J. (2019). Roadmap to 3D-Printed Oral Pharmaceutical Dosage Forms: Feedstock Filament Properties and Characterization for Fused Deposition Modeling. J. Pharm. Sci..

[67] Ilieva S., Georgieva D., Petkova V., Dimitrov M. (2023). Study and Characterization of Polyvinyl Alcohol-Based Formulations for 3D Printlets Obtained via Fused Deposition Modeling. Pharmaceutics.

[68] Wu J., Chen N., Wang Q. (2018). Preparation of novel thermoplastic poly(vinyl alcohol) with improved processability for fused deposition modeling. Polym. Adv. Technol..

[69] Pereira B.C., Isreb A., Forbes R.T., Dores F., Habashy R., Petit J.B., Alhnan M.A., Oga E.F. (2019). ‘Temporary Plasticiser’: A novel solution to fabricate 3D printed patient-centred cardiovascular ‘Polypill’ architectures. Eur. J. Pharm. Biopharm..

[70] Wei C., Solanki N.G., Vasoya J.M., Shah A.V., Serajuddin A.T.M. (2020). Development of 3D Printed Tablets by Fused Deposition Modeling Using Polyvinyl Alcohol as Polymeric Matrix for Rapid Drug Release. J. Pharm. Sci..

[71] Thanawuth K., Sutthapitaksakul L., Konthong S., Suttiruengwong S. (2021). Impact of Drug Loading Method on Drug Release from 3D-Printed Tablets Made from Filaments Fabricated by Hot-Melt Extrusion and Impregnation Processes. Pharmaceutics.

[72] Uboldi M., Chiappa A., Pertile M., Piazza A., Tagliabue S., Foppoli A., Palugan L., Gazzaniga A., Zema L., Melocchi A. (2023). Investigation on the use of fused deposition modeling for the production of IR dosage forms containing Timapiprant. Int. J. Pharm. X.

[73] Crișan A.G., Porfire A., Ambrus R., Katona G., Rus L.M., Porfire A. (2021). Polyvinyl Alcohol-Based 3D Printed Tablets: Novel Insight into the Influence of Polymer Particle Size on Filament Preparation and Drug Release Performance. Pharmaceuticals.

[74] Obeid S., Mad M., Krkobabi M., Ibri S. (2021). Predicting drug release from diazepam FDM printed tablets using deep learning approach: Influence of process parameters and tablet surface/volume ratio. Int. J. Pharm..

[75] Gottschalk N., Quodbach J., Elia A., Hess F., Bogdahn M. (2022). Determination of feed forces to improve process understanding of Fused Deposition Modeling 3D printing and to ensure mass conformity of printed solid oral dosage forms. Int. J. Pharm..

[76] Gioumouxouzis C.I., Katsamenis O.L., Bouropoulos N., Fatouros D.G. (2017). 3D printed oral solid dosage forms containing hydrochlorothiazide for controlled drug delivery. J. Drug Deliv. Sci. Technol..

[77] Tagami T., Nagata N., Hayashi N., Ogawa E., Fukushige K. (2018). Defined drug release from 3D-printed composite tablets consisting of drug- loaded polyvinylalcohol and a water-soluble or water-insoluble polymer filler. Int. J. Pharm..

[78] Goyanes A., Robles P., Buanz A., Basit A.W., Gaisford S. (2015). Effect of geometry on drug release from 3D printed tablets. Int. J. Pharm..

[79] Saviano M., Aquino R.P., Del Gaudio P., Sansone F., Russo P. (2019). Poly(vinyl alcohol) 3D printed tablets: The effect of polymer particle size on drug loading and process efficiency. Int. J. Pharm..

[80] Obeid S., Madzarevic M., Ibric S. (2021). Tailoring amlodipine release from 3D printed tablets: Influence of infill patterns and wall thickness. Int. J. Pharm..

[81] Matijašić G., Gretić M., Kezerić K., Petanjek J., Ema V. (2019). Preparation of Filaments and the 3D Printing of Dronedarone HCl Tablets for Treating Cardiac Arrhythmias. PharmSciTech.

[82] Kozakiewicz-lata M., Junak A., Adrianna Z., Prusik K., Szymczyk-zi P. (2022). Adjusting the melting point of an Active Pharmaceutical Ingredient (API) via cocrystal formation enables processing of high melting drugs via combined hot melt and materials extrusion (HME and ME). Addit. Manuf..

[83] Qijun L., Haoyang W., Danyang J., Xiaoying G., Pan H. (2017). Preparation and investigation of controlled-release glipizide novel oral device with three-dimensional printing. Int. J. Pharm..

[84] Vervaet C., Macedo J., Samaro A. (2020). Processability of poly (vinyl alcohol) Based Filaments with Paracetamol Prepared by Hot-Melt Extrusion for Additive Manufacturing. J. Pharm. Sci..

[85] Đuranovic M., Madzarevic M., Ivkovi B., Ibri S., Cviji S. (2021). The evaluation of the effect of different superdisintegrants on the drug release from FDM 3D printed tablets through different applied strategies: In vitro-in silico assessment. Int. J. Pharm..

[86] Goyanes A., Kobayashi M., Martínez-pacheco R., Gaisford S., Basit A.W. (2016). Fused- filament 3D printing of drug products: Microstructure analysis and drug release characteristics of PVA-based caplets. Int. J. Pharm..

[87] Nukala P.K., Palekar S., Patki M., Patel K. (2019). Abuse Deterrent Immediate Release Egg-Shaped Tablet (Egglets) Using 3D Printing Technology: Quality by Design to Optimize Drug Release and Extraction. PharmSciTech.

[88] Goyanes A., Chang H., Sedough D., Hatton G.B., Wang J., Buanz A., Gaisford S., Basit A.W. (2015). Fabrication of controlled-release budesonide tablets via desktop (FDM). Int. J. Pharm..

[89] Goyanes A., Wang J., Buanz A., Martínez-Pacheco R., Telford R., Gaisford S., Basit A.W. (2015). 3D Printing of Medicines- Engineering Novel Oral Devices with Unique Design and Drug Release Characteristics. Mol. Pharm..

[90] Jamróz W., Kurek M., Ewelina Ł., Szafraniec J., Knapik-kowalczuk J., Syrek K., Paluch M., Jachowicz R. (2017). 3D printed orodispersible films with Aripiprazole. Int. J. Pharm..

[91] Chen D., Xu X., Li R., Zang G., Zhang Y., Wang M., Xiong M., Xu J., Wang T., Fu H. (2020). Preparation and In vitro Evaluation of FDM 3D-Printed Ellipsoid-Shaped Gastric Floating Tablets with Low Infill Percentages. PharmSciTech.

[92] Windolf H., Chamberlain R., Breitkreutz J., Quodbach J. (2022). 3D Printed Mini-Floating-Polypill for Parkinson’s Disease: Combination of Levodopa, Benserazide, and Pramipexole in Various Dosing for Personalized Therapy. Pharmaceutics.

[93] Matija G., Greti M., Vin J., Poropat A., Cuculi L., Raheli T. (2019). Design and 3D printing of multi-compartmental PVA capsules for drug delivery. J. Drug Deliv. Sci. Technol..

[94] Kraisit P., Limpamanoch P., Hirun N., Limmatvapirat S., Filam P.V.A. (2022). Design and development of 3D-printed bento box model for controlled drug release of propranolol HCl following pharmacopeia dissolution guidelines. Int. J. Pharm..

[95] Smith D., Kapoor Y., Hermans A., Nofsinger R., Kesisoglou F., Gustafson P., Procopio A. (2018). 3D printed capsules for quantitative regional absorption studies in the GI tract. Int. J. Pharm..

[96] Charoenying T., Patrojanasophon P., Ngawhirunpat T. (2020). Three-dimensional (3D)-printed devices composed of hydrophilic cap and hydrophobic body for improving buoyancy and gastric retention of domperidone tablets. Eur. J. Pharm. Sci..

[97] Cotabarren I., Gallo L. (2020). 3D printing of PVA capsular devices for modified drug delivery: Design and in-vitro dissolution studies. Drug Dev. Ind. Pharm..

[98] Xu X., Zhao J., Wang M., Wang L., Yang J. (2019). 3D Printed Polyvinyl Alcohol Tablets with Multiple Release Profiles. Nat. Sci. Rep..

[99] Shi K., Salvage J.P., Maniruzzaman M., Nokhodchi A. (2021). Role of release modifiers to modulate drug release from fused deposition modelling (FDM) 3D printed tablets. Int. J. Pharm..

[100] Ibrahim M., Barnes M., Mcmillin R., Cook D.W., Smith S., Halquist M., Wijesinghe D., Roper T.D. (2019). 3D Printing of Metformin HCl PVA Tablets by Fused Deposition Modeling: Drug Loading, Tablet Design, and Dissolution Studies. PharmSciTech.

[101] Skowyra J., Pietrzak K., Alhnan M.A. (2015). Fabrication of extended-release patient-tailored prednisolone tablets via fused deposition modelling (FDM) 3D printing. Eur. J. Pharm. Sci..

[102] Charoenying T., Patrojanasophon P., Ngawhirunpat T., Rojanarata T., Akkaramongkolporn P., Opanasopit P. (2020). Fabrication of floating capsule-in- 3D-printed devices as gastro-retentive delivery systems of amoxicillin. J. Drug Deliv. Sci. Technol..

[103] Varun S., Moinuddin Shaik K., Choudhury A., Kumar P., Kala P. (2021). Investigations of process parameters during dissolution studies of drug loaded 3D printed tablets. J. Eng. Med..

[104] Junqueira L.A., Tabriz A.G., Jos F., Carobini L.R., Pard U., Ant M., Brand F., Douroumis D. (2022). Coupling of Fused Deposition Modeling and Inkjet Printing to Produce Drug Loaded 3D Printed Tablets. Pharmaceutics.

[105] Basa B., Jakab G., Kallai-Szabo N., Borbas B., Fulop V., Balogh E., Antal I. (2021). Evaluation of Biodegradable PVA-Based 3D Printed Carriers during Dissolution. Materials.

[106] Chaudhari V.S., Malakar T.K., Murty U.S., Banerjee S. (2021). Fused deposition modeling (FDM)-mediated 3D-printed mouth-dissolving wafers loaded with nanostructured lipid carriers (NLCs) for in vitro release. J. Mater. Res..

